# Supplementary material for: Have maternal or paternal ages any impact on the prenatal incidence of genomic copy number variants associated with fetal structural anomalies?
Source: PLoS One. 2021 Jul 9;16(7):e0253866. doi: 10.1371/journal.pone.0253866 (PMC8270131; doi:10.1371/journal.pone.0253866)
Supplement: S1 Table — (DOCX) [file pone.0253866.s001.docx]

**S1 Table.**

RECURRENT CNV

| arr[GRCh36] 1q21.1(144974142_146253330)x3 mat |
| --- |
| arr[CRGh37] 1q21.1(145373269-145972481)x1 pat  arr[GRCh36] 3q29(197371607_198438099)x1 dn  arr[CRGh37] 7q11.23 (72228688_73869094)x1  arr[GRCh37] 8p23.1(8111027_11805960)x1 dn  arr[GRCh36] 11q13.2q13.4(67684435_73938053)x1  arr[CRGh 37] 15q13.2q13.3(30931844_32618383)x1 dn  arr[CRGh37] 15q13.3(30635512_30686321)x1  arr[CRGh37] 15q25.2q25.3(84016862-87813407)x1 dn  arr[GRCh36] 16p11.2(29500284_30098069)x1 dn  arr[GRCh36] 16p11.2(29733159_30170522)x3 pat  arr[GRCh37] 16p13.3(3703111_3893389)x3 pat  arr[CRGh37] 17q21.31(43706886_44351152)x1 dn  arr[CRGh37] 17q12(31930168_33174843)x1 dn  arr[CRGh37] 17q12(31930168_33225459)x1  arr[CRGh37] 17q12(34816541_36284013)x1 dn  arr[GRCh36] 22q11.21(17041724_19835417)x1  arr[GRCh36] 22q11.21(17193937_19791811)x1  arr[GRCh36] 22q11.21(17274835_19791811)x1  arr[GRCh36] 22q11.21(17274835_19835417)x1  arr[GRCh36] 22q11.21(17274835_19770514)x1 dn,Xp22.31(6477006_7920118)x1 pat  arr[CRGh37] 22q11.21(18813937_21505417)x1  arr[CRGh37] 22q11.21(19172841_23664737)x1  arr[CRGh37] 22q11.21(19172841_19843647)x1  arr[CRGh37] 22q11.21(19172841_19843647)x1  arr[CRGh37] 22q11.21(19172841_22691547)x1  arr[CRGh37] 22q11.21(19172841_22691547)x1  arr[CRGh37] 22q11.21(18894835_21809009)x3 |

NON-RECURRENT CNV

| arr[GRCh37] 1p36.32p36.22(2456118_11926402)x1 |
| --- |
| arr[GRCh36] 1p36.33p36.32(746956-3618571)x1 |
| arr[GRCh36] 2p21(44879507-45168080)x1  arr[GRCh37] 3p26.2p25.3(3214488_11056870)x1 |
| arr[GRCh36] 3p26.3(233708-1056880)x3 |
| arr[GRCh36] 3q28q29(193302544_199379625)x3, 5p15.33p15.32(75149_4663369)x1 |
| arr[GRCh36] 4p16.3p16.2(81744_3842178)x1, 7p22.3p22.1(141577_6751312)x3 |
| arr[GRCh36] 4q13.3q21.1(74988033_78182594)x1 dn |
| arr[GRCh37] 4q22.3q24(96214396-101668758)x1 |
| arr[GRCh36] 4q26.3q31.22(132054832_146800144)x1, 4q34.1q35.1(174996570_184686470)x1  arr[GRCh37] 8p23.1(8111027_11805960)x3 dn |
|  |
| arr[GRCh37] 8p23.3p11.1(1257325_43735846)x1,8q11.21q24.3(46956592-146166601)x3 |
| arr[GRCh36] 8p23.3p22(183250_18157968)x1 |
| arr[GRCh37] 8q23.3q24.23(113507210-136493214)x1 |
| arr[GRCh37] 8q23.3q24.3(116245451_141711953)x1 |
| arr[GRCh37] 8q24.13q24.3(122729432-146166601)x3 |
| arr[GRCh37] 9p21.1p13.1(31437716_39146954)x2~3,15q13.2q13.3(28719136_30297218)x1 |
| arr[GRCh36] 9p24.3(201086_1655294)x1, 21q22.13q22.3(36929840_46914745)x3  arr[CRGh37] 10q11.22q11.23(46691599_51736421)x1  arr[GRCh36] 11q23.3q25(116478634-134450377)x3,22q11.1q11.21(14884318-18691784)x3  arr[GRCh36] 11q13.5q14.1(75,024,606-77,017,130)x3 dn |
|  |
|  |
| arr[GRCh36] 12p13.33(74808_380559)x1,14q32.2q32.33(98460724_106352979)x3 |
| arr[GRCh37] 13q13.3q14.3(38975816_52429657)x1 |
| arr[GRCh37] 13q33.2q34(106147104_115019701)x1 |
|  |
| arr[GRCh37] 15q26.2q26.3(96724029_99607414)x1 |
| arr[GRCh36] 16q24.1q24.2(84813614_86343934)x1 dn  arr[GRCh37] 17q25.1q25.3(74097498_81044553)x3 |
| arr[GRCh36] 18p11.32p11.21(4316_14112521)x1 |
| arr[GRCh37] 18p11.32p11.21(99191_14076542)x4  arr[GRCh36] 22q12.1(25705542_26888205)x1 dn  arr[GRCh36] 22q13.32q13.33(47244598_49525123)x1 |
| arr[GRCh36] Xp11.23(48193484_48410496)x2 mat |
| arr[GRCh36] Xp22.33(435020_527516)x1 |
| arr[GRCh37] Xq28(152802566-153331874)x2 mat |
| arr[GRCh36]Yp11.32p11.31(1545_2371454)x1,Yq11.221q12(17566412_57277728)x0,Yq12(57512976_57741356) |
|  |
